# CD74 and intratumoral immune response in breast cancer

**DOI:** 10.18632/oncotarget.8610

**Published:** 2016-04-06

**Authors:** Zhi-Qiang Wang, Katy Milne, John R. Webb, Peter H. Watson

**Affiliations:** ^1^ Trev and Joyce Deeley Research Centre, British Columbia Cancer Agency, Victoria, British Columbia, Canada; ^2^ Department of Pathology and Laboratory Medicine, University of British Columbia, Vancouver, British Columbia, Canada

**Keywords:** CD74, MHCII, CD4, CD8, CD68

## Abstract

CD74 (invariant chain) plays a role in MHC class II antigen presentation. We assessed CD74 and MHCII expression in tumor cells, as well as CD8, CD4, and CD68 tumor infiltrating leucocyte (TIL) density by immunohistochemistry in a cohort of 492 breast cancer patients. CD74 expression was associated with poor prognostic markers including patient age, tumor grade, ER status, non-Luminal A subtypes, and with MHCII expression and higher TIL densities, particularly in the Basal-like subgroup. Univariate analysis showed a favorable prognostic effect of CD74 (Hazard ratio = 0.46, 95% CI = 0.26–0.89, *p* = 0.022) and for combined CD74/MHCII (Hazard ratio = 0.26, 95% CI = 0.17–0.81, *p* = 0.014) positive status for overall survival that was only manifested in the Basal-like subgroup. CD74 and MHCII expression is associated with patient survival in Basal-like breast cancer, and the association with TIL may reflect an effective intratumoral immune response.

## INTRODUCTION

CD74 (invariant chain, Li) is expressed by breast tumor cells [1] as well as by several immune cell types. CD74 has dual roles as a component of the MHC class II antigen presentation pathway and as a cytokine receptor [2], as well as intracellular effects on activation of transcription [3, 4]. The MHCII is central to the process of presentation of peptides to CD4 T cells and consequently impacts many aspects of adaptive immunity including activation of effector CD8 T cells [5]. The macrophage inhibitory factor (MIF) is a prevalent cytokine that acts through CD74 to promote cell proliferation, migration and survival pathways in both immune and epithelial cell types [6–8].

CD74 expression in tumor cells might therefore be expected to mediate both pro and anti tumor effects attributable to its cytokine signaling and antigen presenting functions. Several previous studies based on mostly small cohorts have shown that CD74 is associated with ER negative and/or triple negative subgroups, but have been discordant with regard to the prognostic significance of CD74 [9, 10]. Given this dichotomy we set out to examine the relationship between CD74 and outcomes in breast cancer. We hypothesized that tumors that express CD74 along with MHCII, both key components of the antigen presenting machinery, represent those tumors most susceptible to a productive tumor infiltrating leukocyte (TIL) response and correspondingly good outcomes.

## RESULTS

### Cohort characteristics

A total of 492 patients with primary breast cancer diagnosed in the period 1988–1995 were studied. Follow-up outcomes data was available, with mean of 87 months (range 2 to 251 months). There were 195 breast specific deaths (mean time 55 months from diagnosis) and 297 survivors (mean time to last follow-up date 107 months). Primary therapy included surgical resection in all cases followed by adjuvant hormone, radiation, and chemotherapy in 373 (76%), 182 (37%), 102 (21%) cases respectively and 31 (6%) did not receive any form of systemic therapy. The clinical-pathological characteristics of the population are provided (Table 1).

**Table 1:** Clinical-pathological characteristics of the whole cohort

Parameter	Status	Cases	%
Age at diagnosis	≤ 35 years	13	3
	> 35years	479	97
Tumor size^a^	T1a/b	4	1
	T1c	124	25
	T2	277	56
	T3	57	12
	Unknown	30	6
Nodal status	Positive	223	45
	Negative	244	50
	Unknown	25	5
Tumor grade	1	81	16
	2	297	60
	3	111	23
	Unknown	3	1
ER^b^	Positive	250	51
	Negative	242	49
	Unknown	0	0
PR^b^	Positive	250	51
	Negative	242	49
	Unknown	0	0
Molecular subtypes	Luminal A	197	40
	Luminal B	52	11
	Her2	72	15
	TNNB^c^	43	9
	Basal-like	73	15
	Unclassified	55	11

^a^Tumor size: 0.1 cm < T1a/b < 1 cm; 1 cm ≤ T1c < 2 cm; 2 cm ≤ T2 < 5 cm; 5 cm ≤ T3.

^b^ER negative defined as < 10 fmol/mg protein and PR negative as ≤ 15 fmol/mg protein (ligand binding assay).

^c^TNNB=Triple-negative-non-basal.

### Expression of CD74 and association with clinical-pathological features

**Figure 1:**
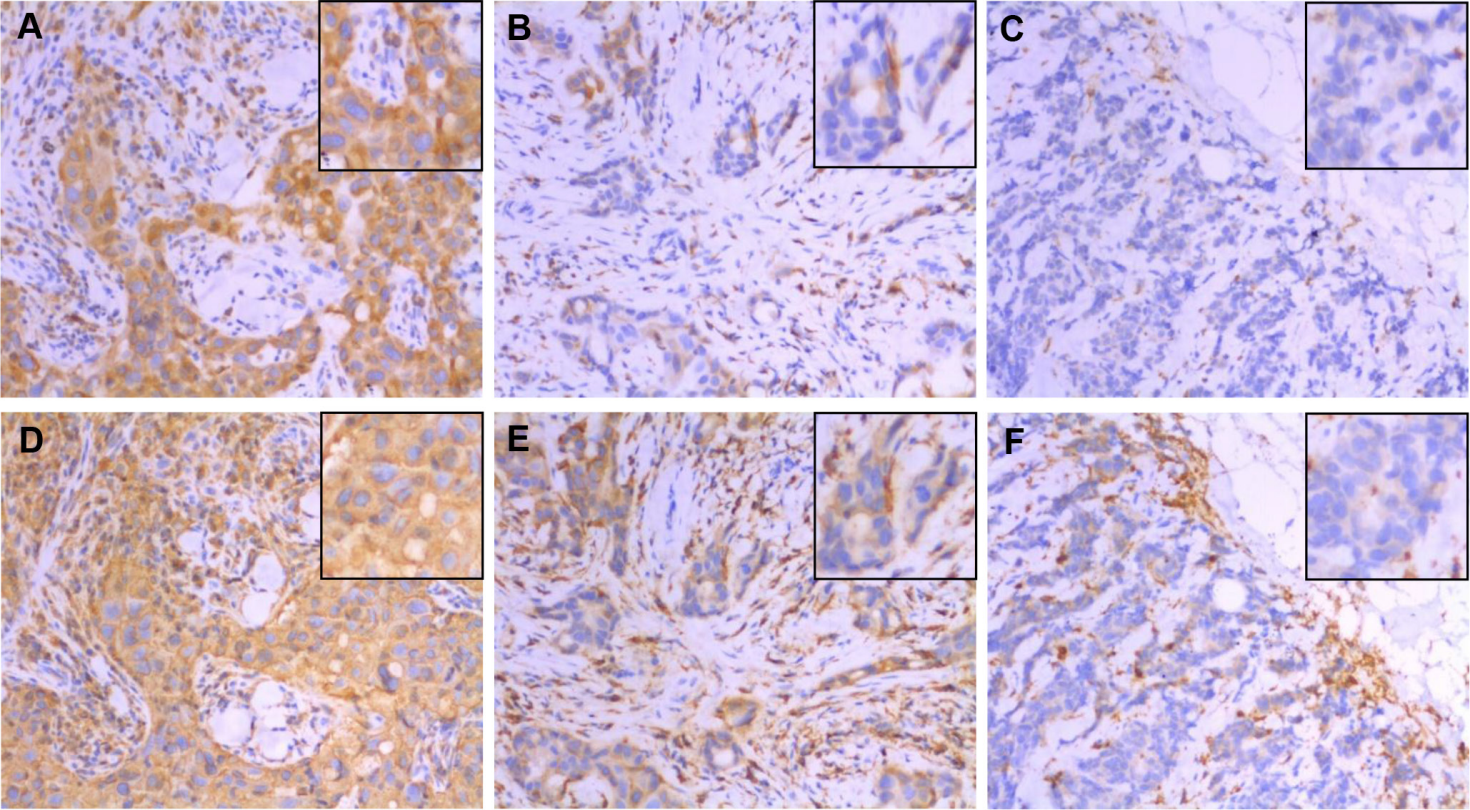
Expression of CD74 (top row) and MHCII (bottom row) in the same areas of three representative tumors as determined by immunohistochemistry These three cases scored as CD74 strong positive / MHCII strong positive (panels **A** and **D**), CD74 weak positive/MHCII weak positive (panels **B** and **E**), and CD74 negative/MHCII negative (panels **C** and **F**) for tumor cell expression. Main images taken at 200× original magnification with insets showing detail within the image. Note that TIL staining positive for CD74 and MHCII are present in stromal and epithelial compartments of all cases but were not included in scoring.

Expression of CD74 within tumor cells showed a predominantly cytoplasmic staining pattern with weak membrane staining visible in occasional cells, and was heterogeneous within positive staining tumors. CD74 expression was present in 139 (28%) cases (Figure 1). CD74 was associated with patient age (*p* = 0.001), tumor grade (*p* = 0.003), and ER status (*p* = 0.006) (Table 2). The frequency of CD74 expression between molecular intrinsic subtype classes was also significantly different, with high levels of CD74 present in a small proportion (21%) of Luminal A subtype tumors but significantly higher proportions (37%–38%) of Luminal B, Triple Negative Non-basal (TNNB), and Basal-like subtype tumors (*p* = 0.009, *p* = 0.022, *p* = 0.003 respectively) (Table 2).

**Table 2:** Association between CD74, MHCII expression and clinical-pathological characteristics

Parameter		CD74 expression			MHCII expression		
		Low (%)	High (%)	*p*-value	Low (%)	High (%)	*p*-value
Age at diagnosis (yrs)	≤ 35 years	4 (31%)	9 (69%)	**0.001**	4 (33%)	8 (67%)	**< 0.0001**
	> 35 years	349 (73%)	130 (27%)		352 (81%)	81 (19%)	
Tumour size (cm)	T1a/b	4 (70%)	0 (0%)	0.555	2 (67%)	1 (33%)	0.195
	T1c	87 (73%)	37 (30%)		65 (88%)	9 (12%)	
	T2	201 (73%)	76 (27%)		223 (78%)	63 (22%)	
	T3	38 (67%)	19 (33%)		41 (79%)	11 (21%)	
Nodal status	Positive	178 (71%)	72 (29%)	0.616	185 (82%)	41 (18%)	0.537
	Negative	179 (73%)	65 (27%)		154 (79%)	40 (21%)	
Tumor grade	1	65 (80%)	16 (20%)	**0.003**	69 (93%)	5 (7%)	**< 0.0001**
	2	220 (74%)	77 (26%)		219 (82%)	47 (18%)	
	3	66 (59%)	45 (41%)		66 (64%)	37 (36%)	
ER status	Positive	249 (76%)	80 (24%)	**0.001**	257 (87%)	37 (13%)	**< 0.0001**
	Negative	104 (64%)	59 (36%)		99 (66%)	52 (34%)	
PR status	Positive	178 (74%)	64 (26%)	0.423	182 (84%)	35 (16%)	0.058
	Negative	175 (70%)	75 (30%)		174 (76%)	54 (24%)	
Molecular subtypes	Luminal A	156 (79%)	41 (21%)		156 (89%)	20 (11%)	
	Luminal B	32 (62%)	20 (38%)	**0.009**	37 (76%)	12 (24%)	**0.020**
	Her2	51 (71%)	21 (29%)		59 (84%)	11 (16%)	
	TNNB^a^	27 (63%)	16 (37%)	**0.022**	22 (65%)	12 (35%)	**0.0004**
	Basal-like	45 (62%)	28 (38%)	**0.003**	39 (57%)	29 (43%)	**< 0.0001**

^a^TNNB = Triple Negative Non-basal.

### Association of CD74 with outcomes

**Figure 2:**
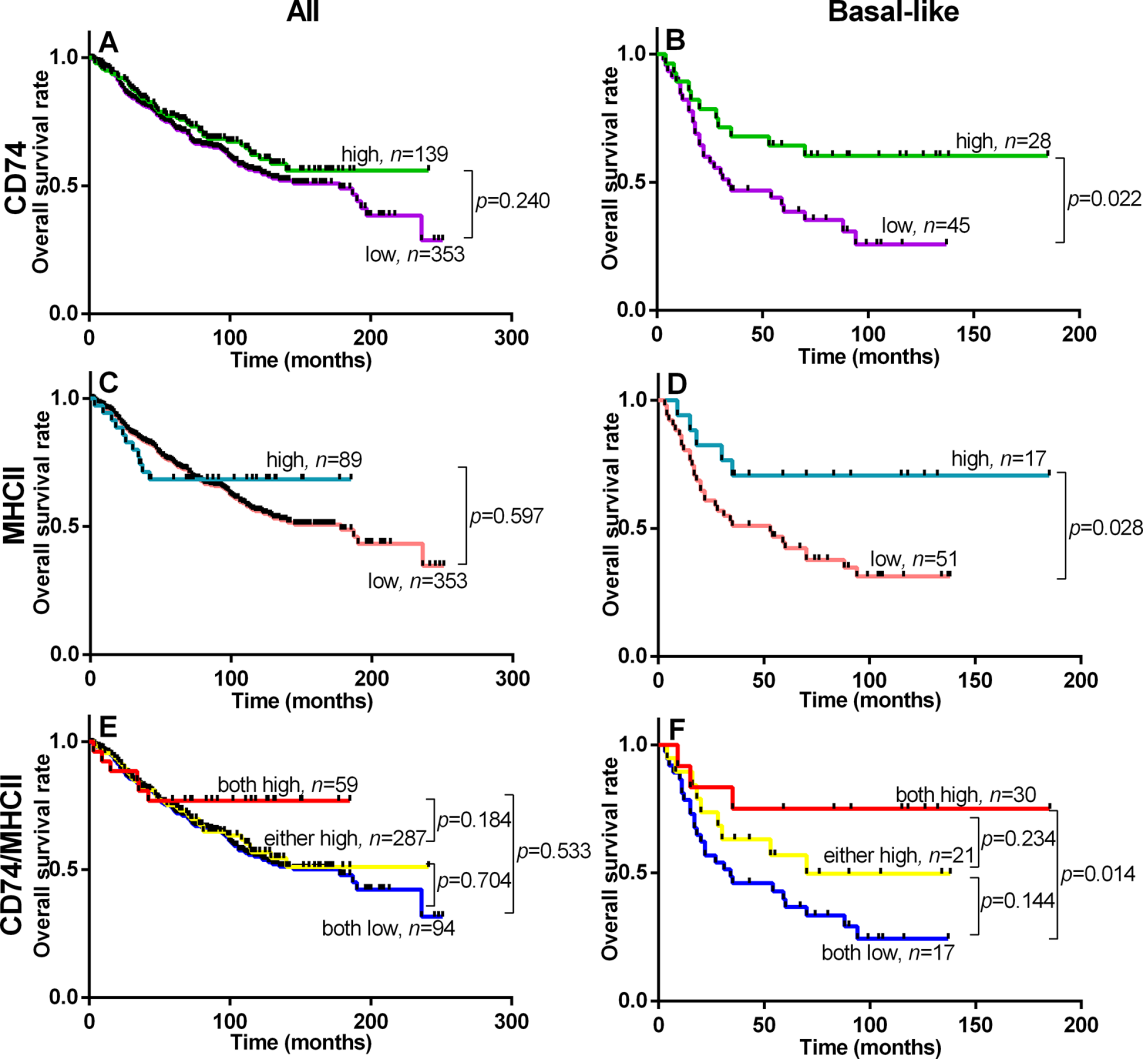
Overall Survival within entire cohort (All, left column) and Basal-like subgroup (Basal-like, right column) relative to status of CD74 (panels A and B), MHCII (panels C and D), and CD74/MHCII combined (panels E and F) Kaplan Meier plots are shown with logrank test *p* values and *n* = total number of subjects within each curve.

Univariate analysis of standard prognostic factors in the entire cohort confirmed patient age, high tumor grade, tumor size, nodal status, ER status and PR status as significant prognostic factors (Supplementary Table 1). Tumor subtype was also strongly prognostic with the rank order of good to poor overall survival subtypes as follows; Luminal A > Luminal B > Her2, TNNB, > Basal-like. CD74 was not prognostic for relapse free survival (RFS) or overall breast cancer specific survival (OS) in the overall cohort. However CD74 was associated with RFS and OS within the Basal-like subset (*p* = 0.018 and *p* = 0.022 respectively) (Figure 2, Table 3). CD74 was not prognostic in other subtypes (including Luminal A and Her2 subsets with larger or comparable subset sizes or Luminal B and TNNB subsets with relatively smaller case numbers). In multivariate analysis of CD74 with clinical prognostic factors within the Basal-like subset, only CD74 was independently prognostic and significant for both RFS and OS (Table 3). In addition, univariate analysis in the Basal-like subgroup showed that CD74/MHCII combined status was independently prognostic and significant for both RFS and OS (Table 4).

**Table 3:** Relapse free survival and overall survival univariate log-rank and cox regression analysis for association of clinical parameters and CD74 or MHCII status in the Basal-like subgroup

A. Recurrence free survival		Univariate		Multivariate	
**Parameter**	**Comparison**	**HR (95% CI)**	***p*-value**	**HR (95% CI)**	***p*-value**
Age at diagnosis (yrs)	> 35 vs ≤ 35	0.71 (0.20–2.20)	0.509	0.31 (0.09–1.11)	0.072
Tumour size (cm)	> 2 cm vs ≤ 2 cm	2.71 (1.02–4.54)	**0.047**	2.95 (0.82–10.67)	0.100
Nodal status	pos vs neg	1.81 (0.90–3.37)	0.101	2.33 (0.99–5.52)	0.054
Tumor grade	2 vs 1	0.39 (0.15–1.41)	0.186	0.43 (0.05–3.68)	0.442
	3 vs 1	1.31 (0.35–4.69)	0.712	1.23 (0.50–3.00)	0.652
MHCII expression	high vs low	0.42 (0.23–1.02)	0.058	0.61 (0.26–1.44)	0.259
CD74 expression	high vs low	0.42 (0.23–0.86)	**0.018**	0.25 (0.08–0.80)	**0.020**
**B. Overall survival**		**Univariate**		**Multivariate**	
**Parameter**	**Comparison**	**HR (95% CI)**	***p*-value**	**HR (95% CI)**	***p*-value**
Age at diagnosis (yrs)	> 35 vs ≤ 35	0.78 (0.24–2.38)	0.638	0.35 (0.10–1.20)	0.095
Tumour size (cm)	> 2 cm vs ≤ 2 cm	1.60 (0.75–3.11)	0.246	1.92 (0.63–5.86)	0.251
Nodal status	pos vs neg	1.75 (0.90–3.16)	0.107	1.82 (0.80–4.14)	0.154
Tumor grade	2 vs 1	0.69 (0.23–2.19)	0.552	0.39 (0.05–3.24)	0.381
	3 vs 1	1.05 (0.31–3.57)	0.944	1.21 (0.51–2.91)	0.665
MHCII expression	high vs low	0.37 (0.23–0.91)	**0.028**	0.82 (0.36–1.86)	0.627
CD74 expression	high vs low	0.46 (0.26–0.89)	**0.022**	0.28 (0.09–0.84)	**0.023**

**Table 4:** Relapse free survival and overall survival univariate log-rank and cox regression analysis for association of clinical parameters and CD74/MHCII combined status in the Basal-like subgroup

A. Recurrence free survival		Univariate		Multivariate	
**Parameter**	**Comparison**	**HR (95% CI)**	***p*-value**	**HR (95% CI)**	***p*-value**
Age at diagnosis (yrs)	> 35 vs ≤ 35	0.79 (0.22–3.01)	0.751	0.81 (0.15–4.34)	0.809
Tumour size (cm)	> 2 cm vs ≤ 2 cm	2.88 (0.95–5.50)	0.070	8.02 (1.01–63.78)	**0.049**
Nodal status	pos vs neg	1.79 (0.78–3.92)	0.155	2.66 (0.95–7.44)	0.062
Tumor grade	2 vs 1	0.51 (0.12–2.82)	0.501	2.72 (0.16–47.62)	0.492
	3 vs 1	1.07 (0.13–8.76)	0.946	1.77 (0.63–4.97)	0.278
CD74+MHCII expression	high vs low	0.29 (0.17–0.90)	**0.031**	0.29 (0.07–1.26)	0.099
**B. Overall survival**		**Univariate**		**Multivariate**	
**Parameter**	**Comparison**	**HR (95% CI)**	***p*-value**	**HR (95% CI)**	***p*-value**
Age at diagnosis (yrs)	> 35 vs ≤ 35	0.77 (0.22–2.86)	0.714	0.84 (0.17–4.18)	0.829
Tumour size (cm)	>2 cm vs ≤ 2 cm	2.36 (0.88–4.72)	0.100	3.73 (0.82–17.05)	0.090
Nodal status	pos vs neg	1.59 (0.72–3.41)	0.193	1.95 (0.75–5.07)	0.172
Tumor grade	2 vs 1	0.56 (0.12–3.22)	0.572	1.68 (0.14–20.55)	0.686
	3 vs 1	1.02 (0.14–7.59)	0.983	1.75 (0.67–4.59)	0.256
CD74+MHCII expression	high vs low	0.26 (0.17–0.81)	**0.014**	0.35 (0.11–1.18)	0.091

**Figure 3:**
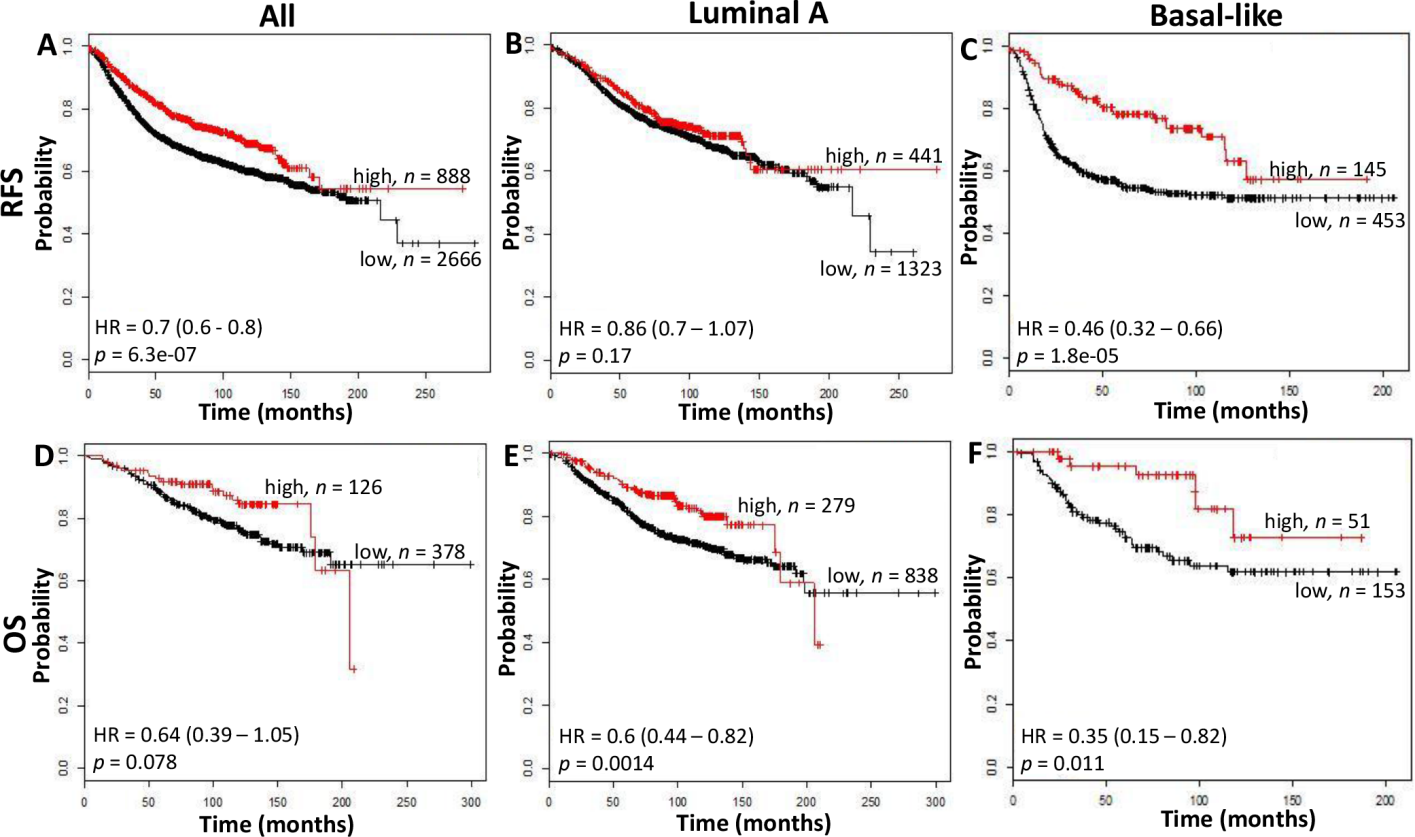
The relation between CD74 and Relapse Free Survival (RFS, top row) and Overall Survival (OS, bottom row) was analyzed in a breast cancer cohort using the kmplotter tool RFS and OS were assessed in all cases (panels **A** and **D**), Luminal A subgroup (panels **B** and **E**), and Basal-like subgroup (panels **C** and **F**) using the Jetprobe set for CD74 (Affymetrix ID: 209619) and a cutpoint at the 75th expression quartile. Kaplan Meier plots are shown with hazard ratios (HR) and logrank test *p* values and *n* = total number of subjects within each curve.

We conducted in-silico analysis of microarray gene expression data using an online survival analysis tool to validate the prognostic effect of CD74 in another cohort [9]. CD74 was prognostic for RFS but not OS within this overall cohort (RFS: *p* < 0.0001, OS: *p* = 0.078) and in the subset containing Basal-like subtype tumors (RFS: *p* < 0.0001; OS: *p* = 0.011), but not in Luminal A tumors (Figure 3) or other subtypes (data not shown).

### Relation between CD74 and MHCII

We next examined the relation of CD74 with MHCII expression. Expression of MHCII within tumor cells showed a predominantly cytoplasmic staining pattern, but with membrane staining visible in some cells, and was relatively homogeneous within positive staining tumors. High expression of MHCII was present in 89 (20%) cases. CD74 expression was closely correlated with MHCII expression in the overall cohort (*p* < 0.0001) and also within all subtypes, and this association was significant in Luminal A (*p* < 0.0001), Luminal B (*p* = 0.0002), Her2 (*p* = 0.011) and Basal-like subsets (*p* = 0.005).

MHCII was also associated with patient age, tumor grade, and ER status (*p* < 0.0001) (Table 2). High levels of MHCII were present in 11% of Luminal A subtype tumors in comparison with significantly higher proportions of Luminal B (24%, *p* = 0.020), TNNB (35%, *p* = 0.0004) and Basal-like subtype tumors (43%, *p* < 0.0001).

### Relation between CD74 and MHCII and intratumoral immune response

**Figure 4:**
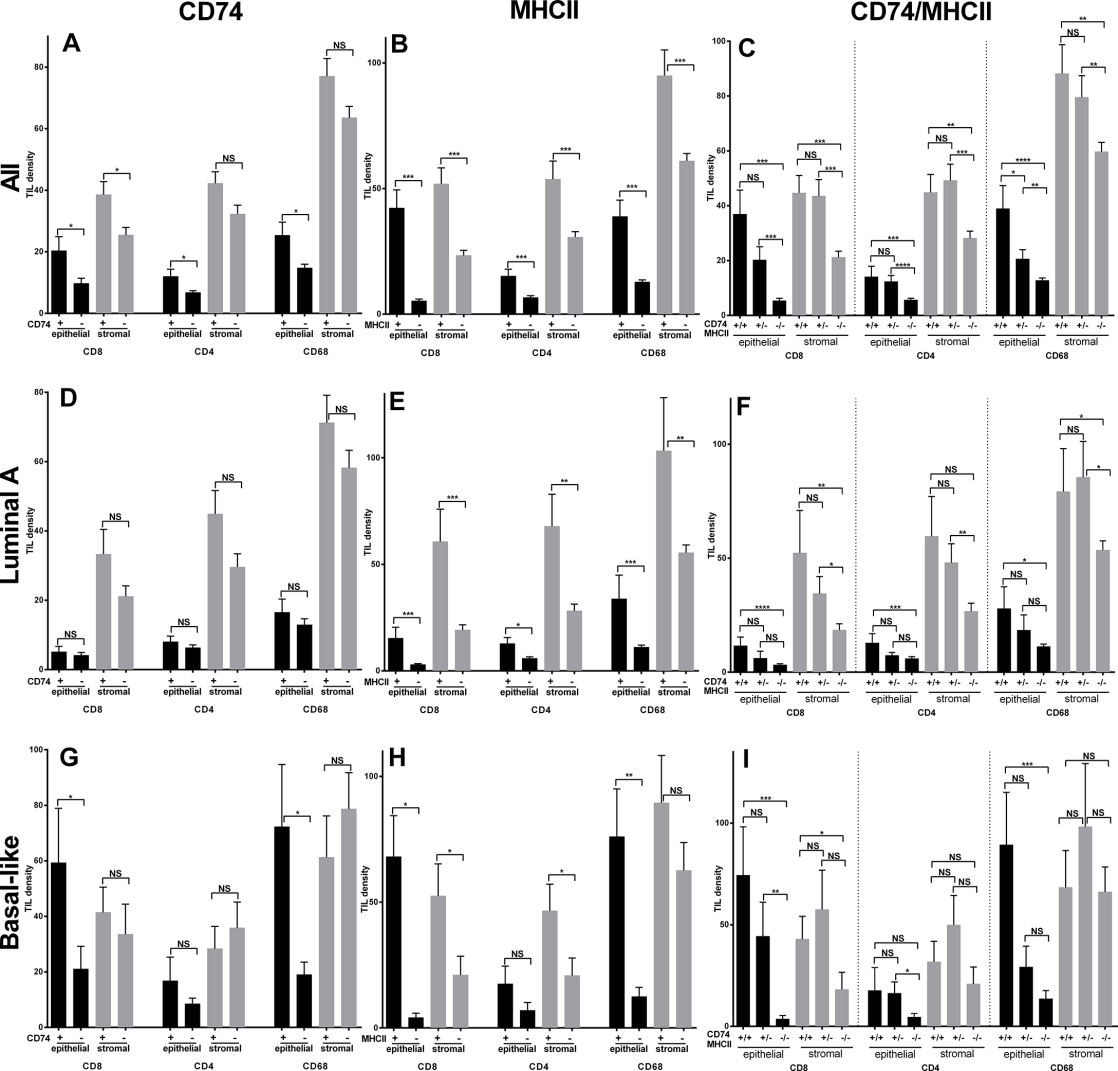
Tumor infiltrating leucocyte (TIL) cell densities in entire cohort (All, top row) and Luminal A subgroup (middle row) and Basal-like subgroup (bottom row) relative to positive and negative status of CD74 (panels A, D, G), MHCII (panels B, E, H), and CD74/MHCII combined (panels C, F, I) Bars represent means +/– standard error. Black and grey bars represent intra-epithelial and intra-stromal densities of CD8, CD4, and CD68 positive TIL respectively. NS = no significance, *p ≤ 0.05, **p ≤ 0.01, ***p ≤ 0.001, ****p ≤ 0.0001.

The intratumoral immune response was assessed by analysis of CD8, CD4, and CD68 infiltrates. In the entire cohort the TIL densities in intra-epithelial versus intra-stromal areas were lower but closely correlated and the mean (standard deviation) densities were as follows; CD8 –12 (33) vs 30 (48), CD4–8 (16) vs 36 (46), CD68–17 (27) vs 66 (59). High levels of CD74 were associated with higher mean densities of CD8, CD4, and CD68 TIL in the entire cohort within both epithelium and stroma, and this was significant for all three TIL types in epithelial areas but only for CD8 in stroma (Figure 4).

Given the well documented differences between Luminal A and Basal-like type tumors with respect to genomic mutational signatures and intratumoral inflammation, and in this study with respect to CD74 and MHCII expression, we focused additional analysis on these two subgroups. Within the Luminal A subset, there were no significant differences in TIL densities between CD74 positive versus negative categories. However in the Basal-like subset of tumors, CD8 and CD68 were significantly higher in CD74 high tumors (*p* < 0.034 and *p* < 0.003) in the epithelial compartment. High levels of MHCII were also associated with higher CD8, CD4, and CD68 TIL and this was statistically significant for epithelium and stromal areas across tumor groups (i.e. all cases), and in both Luminal A, and Basal-like tumors. Further analysis of TIL infiltrates was conducted in relation to dual positive status for both CD74 and MHCII as compared to mixed status and double negative status. Tumors associated with combined CD74/MHCII positive status also showed much higher TIL levels in dual positive compared to mixed or dual negative status tumors across the entire cohort and within Luminal A and Basal-like subgroups (Figure 4). This TIL pattern was mirrored by RFS and OS curves of subgroups with combined positive, mixed, and dual negative status (Figure 2). Analysis of the relation between CD74 and MHCII expression and TIL densities within other subtypes showed comparable trends but only some weak positive associations with respect to individual TIL densities (data not shown).

## DISCUSSION

We have shown that CD74 expression is associated with better prognosis in Basal-like subtype invasive breast cancer. This association correlates with higher levels of MHCII expression by tumor cells and with a dense TIL response.

CD74 is an important component in the functional presentation of MHCII restricted antigens, a key factor in anti-tumor immunity [11–13]. The MHCII complex is stabilized by CD74 in the endoplasmic reticulum allowing subsequent MHCII restricted peptide presentation at the cell surface. It has been shown that CD74 regulates the repertoire of antigens presented by MHCII [14–16] and that this leads to primary engagement of CD4 and then CD8 components of the adaptive immune response, leading to tumor rejection [17–19]. However CD74 is not required for the entire MHCII antigen presenting function and cells lacking CD74 show differences in antigen presentation of only some peptides [20]. The significance in terms of tumor recognition and rejection of this subset of peptides remains to be determined [11, 14]. CD74 can also perform a different function as the receptor for MIF [8]. MIF is a pleiotropic cytokine produced by many cell types that is associated with promotion of tumor growth and invasiveness [21] through effects on both breast epithelial and immune cells [1]. Finally, CD74 can be a target of regulated intramembrane proteolysis by the CatS protease resulting in release of the CD74 intracellular domain. This in turn leads to activation of transcription of chemokines such as CCL2 which is associated with poor prognosis in breast and other cancers [3, 4]. Therefore these different CD74 functions are contradictory with respect to effects on tumor outcome.

Our data here confirms the expected close association between CD74 (Li) and MHCII (HLADR) shown in previous studies [10, 22]. These studies, based on relatively small cohorts (*n* = 52 and *n* = 112 cases) also identified similar relationships between CD74 (and HLADR) and CD3 TIL [22], and high tumor grade. In outcomes analysis that included incorporation of the expression status of HLADM, a second MHCII processing chaperone molecule, co-expression of HLADR/CD74/HLADM conferred a better prognosis than tumors with partial or negative status for all three [10]. However this finding was in contrast to another more recent and larger study showing that associations with poor prognostic factors and in particular with the triple negative subset were observed [9]. This latter association has also been suggested by others [23, 24]. However the Tian study [9] included up to 20% of cases receiving neoadjuvant chemotherapy which may have influenced CD74 expression and complicated outcomes analysis and did not examine correlations with the immune response. Follow-up data was also limited to approximately 5 years. Our current study confirms previous observations that CD74 is closely associated with MHCII, TIL, and several poor prognostic factors. While there was no association with overall survival in the entire cohort, CD74 conferred better outcome in the Basal-like subtype category. Our results therefore suggest that the anti tumor effect predicted to be exerted by CD74's role in facilitating the immune response is dominant over the protumor effect involving MIF signal transduction, expected from laboratory studies on MIF [25–28].

The close association between combined CD74/MHCII expression on tumor cells and the presence of TIL across the entire cohort suggest that a functional relationship exists with antigen presentation on tumor cells. As might be expected, this relationship was present for TIL in general but was strongest for intra-epithelial rather than stromal TIL [29]. TIL assessment has recently emerged as a strong prognostic factor and recent recommendations have focused on scoring stromal TIL. However it should be noted that this is in the context of an ‘immunoscore’ based on H&E stained sections where distinction of intra-epithelial TIL is challenging and TIL subsets cannot be delineated. The role of CD74 in regulating the CCL2 cytokine may also be relevant to the association with TIL, especially macrophages [4, 30].

Dual CD74/MHCII positive status is only positively prognostic in the Basal-like tumor subgroup. One interpretation of our observation is that the functional implication of CD74/MHCII expression in terms of activation of immune responses is common to all subtypes but that tumor susceptibility to the immune system is peculiar to Basal-like tumor cells. The absence of a prognostic significance in the other triple negative non-basal subset may be attributable to the small subgroup size. It should also be noted that while subgroup assignment based only on immunohistochemistry can distinguish prognostic subgroups [31] it has limitations in terms of specificity [32, 33]. While the broad class of triple negative breast tumors is known to be associated with inflammatory infiltrates [34], the triple negative category is very heterogeneous and varies widely in clonal frequency [35]. Tumors of the Basal-like subtype show the most variation in clonal clusters at time of diagnosis, the highest mutation frequency amongst breast intrinsic subtypes, and have the lowest frequency of recurrent mutation in common genes [35, 36]. It might be speculated that these features promote recruitment and activation of multiple T cell clones and contribute to the high TIL density that is a feature of these tumors [37–39]. Both TIL density and immune gene expression signatures have been identified by several groups within Basal-like tumors to correlate with better outcomes [40, 41].

In conclusion, interpretation of our findings should be qualified by the relatively small Basal-like subsets in study and validation cohorts. However, overall these observations are consistent with the view that CD74 expression in tumor cells promotes the intratumoral immune response and is associated with a better prognosis.

## MATERIALS AND METHODS

### Case cohort

A cohort of 492 breast cancer cases was studied representing primary tumors collected by the Manitoba Breast Tumor Bank at time of diagnosis and initial surgical intervention. Age at diagnosis, tumor grade, size, nodal status, and outcomes in terms of relapses, and deaths were recorded [42]. All tumors were histologically classified and graded by one pathologist (PHW). The time of diagnosis and accrual by the bank (1988–1995) predated current biomarker assays. Therefore immunohistochemistry (IHC) was previously performed by the Bank using an auto-immunostainer (Discovery Staining Module, Ventana Medical Systems, AZ, USA) on TMA sections from the cohort for ER, PR, Ki67, CK5/6, EGFR and Her2 biomarkers. ER, PR, and Her2 were scored and positive status assigned according to ACP guidelines. Ki67, CK5/6 and EGFR were also scored and positive status assigned as > 14% (Ki67) or any positive tumor cell staining (CK5/6 and EGFR). On the basis of the IHC determined expression of these five biomarkers the cohort was classified by the Bank into five intrinsic molecular subtypes: Luminal A (ER+/Ki67–/Her–), Luminal B (ER+/Ki67+/Her–), Her2 (Her2+), Triple Negative Non-basal (TNNB, ER–/PR–/Her–/CK5/6–/EGFR–), and Basal-like (ER-/PR-/Her- and either CK5/6+ and/or EGFR+ [31, 43]. The Bank operates with approval of the University of Manitoba Biomedical Research Ethics Board and this research study was conducted under approval from the BC Cancer Agency Research Ethics Board. A report concerning the source of the biospecimens and data used according to the BRISQ guidelines [44] is provided in Supplementary Table 1. In addition, validation studies were performed on a second cohort using an online survival analysis tool based on gene expression and outcomes data from 3554 patients (available at www.kmplot.com) [45].

### Tissue microarray (TMA) construction

Primary tumors were represented in tissue microarrays (TMAs) compiled by the Tumor Bank. To construct a TMA, all cases were initially selected from the database and then sections were re-reviewed to confirm and select areas for coring of corresponding blocks. Duplicate tissue cores (0.6 mm diameter) were taken from central cellular areas of each tumor with a tissue arrayer instrument (Beecher Instruments, Silver Spring, MD, USA).

### Immunohistochemistry and TMA scoring

CD74, MHCII, CD8, CD4, CD68, was also performed on deparaffinized sections from TMAs using a Biocare Medical Intellipath FLX autostainer using reagents from Biocare (Concord, CA) unless otherwise noted. Slides were deparaffinized manually through xylene and graded alcohols then antigen retrieval performed in Biocare's decloaking chamber using Diva decloaking solution for 125°C for 30 seconds. Slides were loaded into the Intellipath FLX, subjected to non-specific blocking with Peroxidased-1 and background sniper then incubated with either CD74, MHCII, CD8, CD4 or CD68 (Supplementary Table 3) in Da Vinci Green diluents for 30 minutes at room temperature. The slides were then incubated with either Mach2 Mouse-(CD74, MHCII, CD8) or Rabbit-(CD4, CD68)-HRP polymer for 30 minutes at room temperature and then detected with IP DAB for 5 minutes followed by counterstaining with a 1:10 dilution of CAT hematoxylin, air drying and coverslipping with Ecomount.

IHC Scoring was performed in a blinded fashion by an experienced breast histopathologist (PHW). Immunostained TMA sections were initially assessed at low magnification to select the core with the highest density of positive cells. The two types of biomarker (CD74 and MHCII expressed by tumor epithelial cells and TIL markers indicating immune cell subsets) were assessed. Only CD74 staining within tumor cells was scored and this could be discriminated as a relatively distinct signal within individual cells and so was assessed by H score method whereby the expression is quantitated as the product of staining intensity (ranked from 0 to 3) and proportion of positive staining tumor cells (0 to 100%) to give an expression score range from 0 to 300; For analysis CD74 tumor cell expression was categorized into low or high expression levels based on the upper quartile (CD74 H scores ≥ 20; corresponding to 1+ intensity in ≥ 20% cells or 2+ intensity in ≥ 10% cells) to avoid any confusion with isolated infiltrating TIL within epithelial areas. Only MHCII staining within tumor cells was scored and this signal was relatively diffuse and so was assessed by assigning an expression score on a 4 point scale (0 to 3+) with 0 = absent, 1 = weak intensity/less than 10% cells, 2 = moderate intensity/10–50% cells, 3 = strong intensity/50–100% cells. As with CD74 expression, in order to avoid any confusion with TIL within epithelial areas, MHCII tumor cell expression status was categorized as low or high based on the upper quartile (MHCII scores (> 1). CD8, CD4, CD68 tumor infiltrating leucocytes (TIL) were assessed as described previously [46] by direct counting up to 20 cells or by estimation when in excess of this number (IHC score, range 0–100) within the selected core area. The area of the entire core occupied by tumor epithelium versus stroma was then assessed followed by estimation of the proportion of positive TIL that were intra-epithelial or intra-stromal (intra-epithelial localization, was defined as lymphocytes within tumor cell nests and/or adjacent to and in direct contact with tumor cells). Intra-epithelial and intra-stromal TIL density per core was then calculated for each type of TIL and for each case.

### Statistical analysis

Associations of CD74 and MHCII with clinical-pathological features were evaluated using Fisher's exact test. Associations of CD74 with TILs were evaluated using *t* test. Survival was calculated using the Kaplan-Meier method and curves were compared with the log-rank test. Multivariate survival analyses were done using Cox regression analysis. All statistical tests were two-sided with significance established at *p*-values less than 0.05. Statistical analyses were performed using Graphpad Prism 6.0 (GraphPad, La Jolla, CA, USA) and SPSS statistics 17 (SPSS, Chicago, IL, USA).

## SUPPLEMENTARY TABLES


