# Supplementary material for: CD74 and intratumoral immune response in breast cancer
Source: Oncotarget. 2016 Apr 6;8(8):12664–74. doi: 10.18632/oncotarget.8610 (PMC5355043; doi:10.18632/oncotarget.8610)
Supplement: Supplementary file 1 [file oncotarget-08-12664-s001.pdf]

CD74 and intratumoral immune response in breast cancer

Supplementary Materials

Supplementary Table S1: Relapse free survival and overall survival univariate log-rank and cox regression analysis for association of clinical parameters and CD74 or MHCII status in all cases

A. Recurrence free survival		Univariate		Multivariate	
**Parameter**	**Comparison**	**HR (95% CI)**	***p*-value**	**HR (95% CI)**	***p*-value**
Age at diagnosis (yrs)	> 35 vs ≤ 35	0.46 (0.22–0.92)	**0.025**	0.47 (0.20–1.10)	0.082
Tumour size (cm)	> 2 cm vs ≤ 2 cm	2.15 (1.42–2.58)	**< 0.0001**	1.99 (1.30–3.04)	**0.001**
Nodal status	pos vs neg	1.99 (1.52–2.65)	**< 0.0001**	2.07 (1.47–2.92)	**0.000**
Tumor grade	2 vs 1	1.47 (0.98–2.04)	0.067	0.87 (0.46–1.66)	0.674
	3 vs 1	1.78 (1.23–2.73)	**0.013**	1.47 (0.93–2.33)	0.102
ER status	pos vs neg	0.44 (0.30–0.53)	**< 0.0001**	0.61 (0.36–1.05)	0.075
PR status	pos vs neg	0.44 (0.33–0.56)	**< 0.0001**	0.59 (0.39–0.89)	**0.012**
Molecular subtypes	Luminal A vs Basal-like	0.29 (0.17–0.48)	**< 0.0001**	1.63 (0.80–3.36)	0.182
	Luminal B vs Basal-like	0.68 (0.41–1.10)	0.121	1.01 (0.45–2.25)	0.986
	Her2 vs Basal-like	1.20 (0.78-–1.85)	0.401	0.49 (0.25–0.95)	**0.036**
	TNNB[a] vs Basal-like	0.80 (0.47–1.39)	0.444	1.51 (0.75–3.02)	0.248
CD74 expression	high vs low	0.82 (0.61–1.10)	0.175	0.71 (0.47–1.07)	0.105
MHCII expression	high vs low	0.89 (0.52–1.53)	0.691	0.81 (0.52–1.28)	0.375

B. Overall survival		Univariate		Multivariate	
**Parameter**	**Comparison**	**HR (95% CI)**	***p*-value**	**HR (95% CI)**	***p*-value**
Age at diagnosis (yrs)	> 35 vs ≤ 35	0.46 (0.10–0.93)	**0.007**	0.61 (0.25–1.51)	0.285
Tumour size (cm)	> 2 cm vs ≤ 2 cm	1.77 (1.19–2.36)	**0.003**	1.78 (1.13–2.80)	**0.012**
Nodal status	pos vs neg	1.83 (1.36–2.46)	**< 0.0001**	1.86 (1.29–2.67)	**0.001**
Tumor grade	2 vs 1	1.52 (0.97–2.16)	0.071	0.71 (0.44–1.14)	0.363
	3 vs 1	2.41 (1.47–3.60)	**0.0003**	1.38 (0.87–2.21)	0.175
ER status	pos vs neg	0.32 (0.20–0.37)	**< 0.0001**	0.52 (0.29–0.92)	**0.025**
PR status	pos vs neg	0.36 (0.26–0.47)	**< 0.0001**	0.59 (0.37–0.94)	**0.026**
Molecular subtypes	Luminal A vs Basal-like	0.24 (0.07–0.20)	**< 0.0001**	1.48 (0.72–3.03)	0.284
	Luminal B vs Basal-like	0.50 (0.30–0.80)	**0.005**	0.26 (0.13–0.53)	**0.000**
	Her2 vs Basal-like	0.99 (0.65–1.51)	0.955	0.58 (0.28–1.24)	0.160
	TNNB[a] vs Basal-like	0.74 (0.44–1.27)	0.283	1.21 (0.60–2.43)	0.593
CD74 expression	high vs low	0.82 (0.61–1.13)	0.240	0.71 (0.46–1.10)	0.122
MHCII expression	high vs low	0.85 (0.49–1.51)	0.597	0.83 (0.51–1.33)	0.436

[a]TNNB = Triple Negative Non-basal.

Supplementary Table S2: A report concerning the source of the biospecimen and data using the BRISQ guidelines [44]

BRISQ Report ID : 40
**Profile**<br>Study Name: CD74<br>Biobank Name: PWATSON<br>Datetime Created: 10-Dec-2015 9:09 pm
**Pre-Acquisition**<br>Biospecimen Type: Tissue (not otherwise specified)<br>Anatomical Site: breast<br>Disease Status of Participants: primary cancer specimens from breast cancer patients<br>Clinical Characteristics Of Participants: invasive breast cancer<br>Vital State Of Participants: alive<br>Disease State: outcomes data provided by MBTB bank<br>Cause of Death: n/a<br>Agonal State: n/a<br>Diagnosis: invasive breast cancer<br>Clinical: primary breast cancer<br>Pathology: invasive carcinoma, NOS<br>Time Between Diagnosis And Sampling: typical pathology service times, 0.5–2 hrs<br>Exposures: none<br>Reproductive Status: unknown<br>Patient Demographic Information: female<br>Accrual Scheme: tumor bank generalized collection scheme....all available breast cancer specimens<br>Nature Biobanking Institutions: Manitoba Breast Tumor Bank
**Acquisition**<br>Collection Mechanism : surgical resection specimens<br>Time From Cessation Blood Flow *In Vivo* To Biosepecimen Excision: unknown<br>Time From Biospecimen Excision Acquisition To Stabilization: typical pathology service times, 0.5–2 hrs<br>Temperature Between Biospecimen Excision Acquisition and Stabilzation: RT<br>Collection Container : unknown
**Stabilization and Preservation**<br>Mechanism of Stabilization: frozen specimens, prior to conversion to FFPE blocks<br>Type of Long Term Preservation : formalin fixed paraffin embedded<br>Constitution of Preservative: 10% neutral buffered formalin<br>Time in Fixative Preservation Solution: unknown<br>Temperature During Time in Preservation Solution: unknown<br>Aliquot Volume: n/a<br>Specimen Size: typical clinical blocks
**Storage and Handling**<br>Storage Temperature: room temp<br>Storage Duration: up to 20 yrs<br>Storage Details: unknown<br>Type of Storage Container: unknown<br>Type of Slide: charged glass for IHC<br>Shipping Parameters: unknown<br>Shipping Duration: unknown<br>Type of Transport Container: unknown<br>Shipping Temperature: room temp<br>Freeze Thaw Parameters:<br>Number of Freeze Thaw Cycles: n/a<br>Duration of Thaw Events: All FFPE blocks created from previously frozen specimens by immersion of frozen aliquot in formalin<br>Time from Last Thaw to Processing: unknown

**Quality Assurance**<br>Composition Assessment and Selection: detailed histology composition analysis of section<br>Gross and Microscopic Review: comprehensive; conducted by a single pathologist<br>Proximity to Primary Pathology of Interest: tumor central portion<br>Method of Enrichment for Relevant Component: TMA coring, targeting central region of tumor in block<br>Details of Enrichment for Relevant Component: n/a<br>Quality Assurance Measures: n/a<br>Embedding reagent/medium: unknown

Supplementary Table S3: Antibodies used for immunohistochemical analysis indicating sources, dilutions used and scoring definitions

Antibody	Source	Host (clone)	Dilution	Definition of positivity
CD74	Abcam	Mouse monoclonal (LN2)	1:100	≥ 20 H score value (intensity 0–3 × % positive cells)
MHCII	Affinity Bioreagents	Mouse monoclonal (CR3/43)	1:500	> 1 (moderate/strong intensity/≥ 10% cells)
CD8	Cell Marque	Mouse monoclonal (C8/144B)	1:250	≥ Median (intra-epithelial or intra-stromal cell score)
CD4	Abcam	Rabbit monoclonal (EPR6855)	1:250	≥ Median (intra-epithelial or intra-stromal cell score)
CD68	Spring Biosciences	Rabbit monoclonal (SP251)	1:150	≥ Median (intra-epithelial or intra-stromal cell score)
